# Renal epithelial cells can release ATP by vesicular fusion

**DOI:** 10.3389/fphys.2013.00238

**Published:** 2013-09-19

**Authors:** Randi G. Bjaelde, Sigrid S. Arnadottir, Morten T. Overgaard, Jens Leipziger, Helle A. Praetorius

**Affiliations:** Department of Biomedicine, Aarhus UniversityAarhus, Denmark

**Keywords:** vesicles, ATP, flow, hypotonic swelling, Ca^2+^, MDCK

## Abstract

Renal epithelial cells have the ability to release nucleotides as paracrine factors. In the intercalated cells of the collecting duct, ATP is released by connexin30 (cx30), which is selectively expressed in this cell type. However, ATP is released by virtually all renal epithelia and the aim of the present study was to identify possible alternative nucleotide release pathways in a renal epithelial cell model. We used MDCK (type1) cells to screen for various potential ATP release pathways. In these cells, inhibition of the vesicular H^+^-ATPases (bafilomycin) reduced both the spontaneous and hypotonically (80%)-induced nucleotide release. Interference with vesicular fusion using N-ethylamide markedly reduced the spontaneous nucleotide release, as did interference with trafficking from the endoplasmic reticulum to the Golgi apparatus (brefeldin A1) and vesicular transport (nocodazole). These findings were substantiated using a siRNA directed against SNAP-23, which significantly reduced spontaneous ATP release. Inhibition of pannexin and connexins did not affect the spontaneous ATP release in this cell type, which consists of ~90% principal cells. TIRF-microscopy of either fluorescently-labeled ATP (MANT-ATP) or quinacrine-loaded vesicles, revealed that spontaneous release of single vesicles could be promoted by either hypoosmolality (50%) or ionomycin. This vesicular release decreased the overall cellular fluorescence by 5.8 and 7.6% respectively. In summary, this study supports the notion that spontaneous and induced ATP release can occur via exocytosis in renal epithelial cells.

## Introduction

P2 receptor activation substantially influences the overall function of renal epithelia. In general, P2 receptor activation dampens transepithelial transport. For example extracellular P2 receptor agonists reduce the HCO^−^_3_ reabsorption in the proximal tubule (Bailey, 2004), and P2Y_2_ receptor activation markedly restrains the arginine vasopressin (AVP)-induced water permeability in the collecting duct (Kishore et al., 1995). Moreover, P2Y_2_ receptor activation has also been shown to impair the activity of epithelial Na^+^ channels (ENaC) in the collecting duct (Pochynyuk et al., 2010). The two mechanisms in the collecting duct underlie the suggested distal hyper-reabsorption documented in P2Y_2_ receptor-deficient mice (Pochynyuk et al., 2010), which also have been associated to the hypertension observed in these animals (Rieg et al., 2007, 2011). Recently, basolateral application of ATP has been shown to significantly inhibit the transepithelial transport in the thick ascending limb via activation of P2X receptors (Silva and Garvin, 2009; Marques et al., 2012). This general pattern of P2 receptor-mediated transport inhibition prompts the suggestion that epithelial ATP release, and the subsequent P2 receptor activation may constitute a negative feedback system that protects renal epithelial cells from overstimulation. This hypothesis should be considered in the light of the type of stimuli known to induce ATP release from renal epithelial cells. These include many types of mechanical perturbations, such as osmotic stress (Boudreault and Grygorczyk, 2004), flow-dependent force on the primary cilium (Praetorius and Leipziger, 2009) and pressure-induced stretching of the epithelium (Praetorius et al., 2004a; Jensen et al., 2007). Moreover, AVP has been shown to induce the release of ATP as detected using a biosensor (Odgaard et al., 2009). It is possible to speculate that the release of ATP from renal epithelia is a self-protection mechanism to avoid either mechanical or hormonal overstimulation of the renal epithelial cells.

The mechanism for regulated ATP release is still only settled for a subset of renal epithelial cells. With the existing data, it is unlikely that there is a single common pathway for ATP release from all types of renal epithelial cells. A recent study demonstrated the importance of connexin30 (cx30)-hemichannels as a pathway for ATP release in the intercalated cells of the murine collecting duct (Sipos et al., 2009), where cx30 is expressed exclusively in the apical domain (McCulloch et al., 2005). As at least some ATP-release pathways are cell type-specific, it would be reasonable to assume that, in general, renal epithelia use several pathways to release this paracrine factor. Other epithelia have been shown to possess cell type-specific ATP release mechanisms, such as the respiratory epithelium where a pore-mediated (pannexin 1) release mechanism predominates in ciliated epithelial cells (Seminario-Vidal et al., 2011) and exocytosis/vesicular release is responsible for the ATP release from the goblet cells (Jones et al., 2012).

Here, we show that both the constitutive and stimulated ATP release by MDCK cells are reduced by interfering with either vesicular acidification or vesicular release, whereas inhibitors of connexins/pannexins did not. Moreover, using total internal reflection fluorescence (TIRF) microscopy we detected the spontaneous and stimulated release of vesicles, which could take up quinacrine and N-methylanthraniloyl-ATP (MANT-ATP), and the content of ATP in the quinacrine-loaded vesicles was confirmed by fluorescence activated cell sorting (FACS) and luminometry. Based on our findings, we conclude that ATP can be released via exocytosis from renal epithelial cells.

## Materials and methods

### Cell culture

Wild-type Madin-Darby Canine Kidney (MDCK) type 1 cells (passages 54–70; American Type Culture Collection, Rockville, MD, USA) were cultured to confluence in Dulbecco's modified Eagle medium (DMEM) with 10% fetal bovine serum, 2 mM glutamine, 100 U ml^−1^ penicillin, and 100 μg ml^−1^ streptomycin (Gibco, Grand Island, NY, USA), but without riboflavin and phenol red as previously described (Praetorius and Spring, 2001; Praetorius et al., 2004b). For microscopy the cells were cultured on 25 mm diameter cover slips (VWR, Herlev, Denmark, for wide field microscopy or 1001/25 (Glaswarenfabrik Karl Hecht KG, Sondheim, Germany) for TIRF microscopy to either confluence or non-confluence depending on the protocol. For all other experiments, MDCK cells were cultured to confluence on 25-mm diameter filter inserts (0.4 μm) HD PET membrane (high pore density polyethylene terephthalate), in 6-well plates, in 25 cm^2^ Falcon tissue culture flasks or in 100 mm petri dishes (all from Becton Dickinson Labware Europe, Le Pont de Claix, France).

### Microscopy and perfusion

MDCK cell mono-layers cultured on coverslips were viewed at 37°C on the stage of an inverted microscope (TE-2000, Nikon) equipped with differential interference contrast (DIC) combined with low-level fluorescent light provided by a xenon lamp and monochromator (Visitech International, Sunderland, UK). Imaging was performed using either a plan Fluo 20X, 0.75 NA or a 60X, 1.4 NA Plan Apo objective (Nikon), an intensified SVGA charged coupled device (CCD) camera and imaging software (Quanticell 2000/Image Pro, VisiTech). The cellular fluorescence was sampled at the rate of 1 Hz and measurements were initiated 60 s prior to the start of perfusion. We used TIRF microscopy to visualize vesicles just beneath the plasma membrane. The TIRF set-up was provided by Bio-Science ApS, Gilleleje, Denmark and consisted of an iMIC stage (Till-Photonics, Munich, Germany) equipped with three lasers (405 nm iWave Toptica, 488 nm iWave Toptica (Toptica Photonics AG, Graefelfing, Germany), and 532 nm Cobolt Jive (Cobolt AB, Solna, Sweden). A Yanus scan-head combined with the Polytrope imaging-mode switch the laser beams via a vanometric mirror so that they focused on the back focal plane of the objective. The lasers were adjusted to an angle of ~64° to create an evanescence field around the glass-salt solution interface. The preparation was imaged with a 60X, 1.45 NA Plan Apo (Olympus) objective and a CCD camera (Sensicam qe, PCO, Kelheim, Germany). The cells were mounted in a semi-open (covered by only half a cover-slip) chamber modified from the chamber (RC-21BRFS) available from Warner Instruments, Hamden, CT, USA. The semi-open chamber avoids the build-up of pressure in the system, reduces evaporation compared to a completely open chamber and retains the good optical properties of a closed chamber. The solutions were superfused at constant flow rates of 12 μl s^−1^, which corresponds to a bulk flow velocity of 820 μm s^−1^ equaling 0.103 dynes cm^−2^ (assuming 6.97 × 10^−3^ poise as the dynamic viscosity of water at 37°C). All antagonist and agonist solutions were prepared from frozen stock-solutions immediately prior to each experiments.

### Analysis of [Ca^2+^]_i_ imaging

On average, 300 fluo-4 loaded MDCK cells were imaged in time-lapse (60 images at 1 Hz), and the cells that displayed spontaneous increases in the fluorescence intensity were identified as follows: the fluorescence intensity of each pixel in the first frame of the image sequence was multiplied by 1.05 and subsequently subtracted from the entire sequence. Using this procedure, cells that displayed an increase in florescence intensity greater than 5% were observed as bright areas on a black background. These bright areas were marked as regions of interest (ROI) in all of the frames in the modified sequence. Subsequently, the ROIs were transferred to the original image sequence, and the average fluorescence intensities for the ROIs were extracted from this sequence. The data were imported to the analysis program Igor Pro (Wavemetics, Lake Oswego, OR, USA) and the multi-peak finding feature of the program was used to identify and quantify each computed Ca^2+^ event. Thus, a Ca^2+^ event was defined as a single increase in fluo 4 fluorescence intensity greater than 5%. For each experiment, his procedure was used to determine the number of responding cells, the number of [Ca^2+^]_i_ events per second in the field of observation (~300 cells), as well as the amplitude and duration of the each event.

### siRNA-mediated knockdown of canine SNAP-23

The protocol used was as previously described by Ge et al. (2003) with minor modifications. Briefly, confluent MDCK cells were re-suspended in serum-free RPMI-1640 medium (Sigma-Aldrich) at a cell density of approximately 5 × 10^6^ ml^−1^. A scrambled siRNA or a siRNA directed against canine SNAP-23 (scrambled control and SASI_Mm01_00176533 and scrambled control from Sigma) or TE-buffer as a control (mock transfection) were added to the cell suspensions, at a final concentration of 2 μ M. The mixtures were incubated on ice for 5 min before the cells were transferred to a 4 mm Gene Pulser cuvette (Bio-Rad-Laboratories, Copenhagen, Denmark) and subjected to a single electroporation pulse (400 V and 975 μ F, Gene Pulser Xcell (Bio-Rad). Immediately after the pulse, 500 μ l DMEM containing 10% fetal bovine serum was added. Each group of cells was divided into three subgroups for analysis at 24, 48, or 72 h. The effectiveness of the siRNA silencing was investigated using quantitative PCR (see below), immunoblotting (see below) and live cell [Ca^2+^]_i_ imaging (see above) for all three time groups.

### Quantitative RT-PCR

The RNeasy MiniKit from QIAGEN GmbH (Hilden, Germany) was used to isolate RNA according to the vendor's protocol. The RNA concentration was determined by spectrophotometery. RNA (50 ng μ l^−1^) was incubated with 0.33 μ M Random Decamers (MWG Biotech, Ebersberg, Germany) for 3 min at 85°C, then the reverse transcriptase reagents (0.46 mM dNTP [TaKaRa Bio Inc., Shiga, Japan], 4.25 μ l of 5x First Strand Buffer [Invitrogen], 4.76 U l^−1^ SuperScript III Reverse Transcriptase [Invitrogen] and 0.24 U l^−1^ SUPERase-ln [Ambion, Austin, TX, USA]) were added. As negative controls, water was added instead of reverse transcriptase or RNA. The reverse transcriptase program was as follows: 5 min at 55°C, 60 min at 45°C, 15 min at 70°C, and 10 min at 4°C. Reverse transcription was confirmed by electrophoresis on a 2% agarose gels (Low EEO; AppliChem GmbH, Darmstadt, Germany) using Tris-borate-EDTA (*TBE*) buffer. Qualitative RT-PCR (qPCR) was performed to determine the relative mRNA levels. The primers used were: SNAP-23-F 5′-GCA TAG AAG AAG GCA TGG AC-3′ (100 nM), SNAP-23-R, 5′-GTT GTT GAG GCT GCC CAT TT-3′ (500 nM), G3PDH-F, 5′-CAC GGC AAA TTC CAC GGC ACA G-3′ (500 nM) and G3PDH-R, 5′-ATG ACC ACC GTC CAT GCCA A-3′ (100 nM). The probes used were; SNAP-23-P 5′-CAT GGG GAG ATG GTG AAG ACA ACT-3′ and G3PDH-P 5′-TTG TCA GCA ATG CCT CCT GCA CCA CCA ACT (both 100 nM, 5′ Fluorescein, 3′ Blackhole Quencher 1). The master mix consisted of: 0.2 mM dNTPs (Invitrogen), 1x Ex Taq buffer (TaKaRa), 5 mM MgCl_2_, 0.025 U Taq μl^−1^ (TaKaRa). The cDNA was diluted two times and the PCR program (1 cycle of 10 min at 95°C; 40 cycles of 30 s at 95°C, 1 min at 60°C, and 1 min 72°C) was run on a Mx3000, Stratagene thermocycler (Agilent Technologies inc., Santa Clara, CA, USA). The data were analyzed using MxPro ver. 4.0 software (Stratagene).

### Protein purification

For protein purification, MDCK cells were resuspended in lysis-buffer (50 mM Tris-HCl, 250 mM NaCl, 0.5% nonyl phenoxypolyethoxylethanol-40, 5 mM EDTA, 20 mM NaF, 0.5 M Phenylmethanesulfonyl fluoride [dissolved in EtOH], phosphatase inhibitor cocktail mix II [diluted 1:100, Sigma], and one protease inhibitor tablet [Roche]), incubated on ice for 15 min with a gentle vortexing every 2 min and then centrifuged for 15 min at 14200 g. The supernatant was transferred to a new tube and the pellet was re-suspended in 500 μl lysis-buffer and stored at −80°C. As control, a piece of fresh mouse kidney was homogenized and treated as described above using lysis-buffer. After 30 min incubation on ice, with intermittent gentle vortexing, the tissue lysate was centrifuged at 14,200 *g* for 30 min, the supernatant was transferred to a new tube, and the pellet was re-suspended in lysis buffer and stored at −80°C. The protein concentrations of the lysates were determined using the Pierce BCA, Protein Assay Kit (Thermo Scientific, Rockford, IL, USA) according to the manufacturer's protocol.

### SDS-PAGE and western blotting

Protein samples (10 μg) were loaded on two identical ready gels (Bio-Rad) and electrophoresed at 125 V performed for 1–1.5 h at room temperature. BenchMark Pre-Stained Protein Ladder (Invitrogen) or Spectra Multicolor Broad Range Protein Ladder (Fermentas, Burlington, Ontario, Canada) were used as molecular weight markers. The proteins were transferred onto ethanol-activated Immobilion-FL PVDF membranes (pore size 0.45 μm, Millipore, Billerica, MA, USA) at 100 V for 1 h at 4°C. After overnight blocking at 4°C with 2% skimmed-milk (ARLA, Viby, Denmark) in 0.1 M PBS, the first membrane was incubated with primary antibody against SNAP-23 (1:1000, Synaptic Systems, Goettingen, Germany) diluted in 0.1 M PBS containing 0.1% Tween-20 (PBS-T) for 1 h at room temperature. As a control, the second membrane was incubated with SNAP-23 primary antibody (1:1000), with SNAP-23 control peptide (1:1000 Synaptic Systems, Goettingen, Germany). The membranes were then incubated with Donkey-anti-rabbit IRDye680 secondary antibody (1:12000, LI-COR GmbH, Bad Homburg, Germany) in the dark, and the bands were visualized using a LI-COR Odyssey scanner.

### Hypotonic stress assay

The MDCK cells were cultured to confluence on 25-mm diameter filter inserts, which allowed samples to be taken both from the apical and basolateral sides of the epithelium. The cells were equilibrated in HEPES buffered salt solution (HBS) for 1 h at 37°C prior to the experiment. At the end of this incubation time, samples for the baseline ATP release were carefully taken and replaced with fresh HBS. Hypotonic stress was induced by replacing half of the HBS on the basolateral side of the filters with water. After 10 min at 37°C, samples were carefully taken from both sides of the filter. All samples were boiled for 1 min immediately after sampling (to prevent potential enzyme dependent ATP degradation) and stores on ice before analysis.

### Isolation of intact vesicles from MDCK cells

For isolation of intact vesicles we used a cell cracker developed at European Molecular Biology Laboratory (Heidelberg, Germany) with an 8.01 mm diameter ball. The cell cracker mechanically disrupts the cells and releases the contents of the cells. The cell cracker was kept on ice for the entire protocol. The MDCK cells loaded with quinacrine (5 μM, 30 min) were passed through the cell cracker 20 times and then briefly centrifuged and re-suspended (repeated 10 times); at this time a significant amount of free quinacrine-loaded vesicles could be observed in the suspension by wide field microscopy (60X, 1.4 NA Plan Apo objective [Nikon]). After the last centrifugation step, the supernatant containing the vesicles was transferred to a new tube and fluorescence-activated cell sorting (FACS) was used to sort the cell debris into four populations, one of which contained only small vesicles (defined by the size and 488 nm fluorescence). A high K^+^ solution (pH 7.2, 125 mM KCl, 0.8 mM MgSO_4_, 14 mM Na-HEPES, 5.6 mM *D*-glucose) was used as the re-suspension media during cell cracking to mimic the cytosolic environment. Cell sorting was performed at the FACS Core Facility, Health, Aarhus University, Denmark using a FACSAria III (Becton Dickinson) high speed cell sorter equipped with a 488 nm laser and a (530/30 nm) emission filter. The vesicles were sorted on the basis of the 488 nm fluorescence, and then size gated to include only the smallest quinacrine positive particles. After sorting, an aliquot of the sample was taken for microscopy and the reminder was centrifuged using an Air-Driven Ultracentrifuge (Airfuge, Beckman) running at 20 psi (corresponds to 100,000 g) for 10 min. The vesicle pellet was re-suspended in lysis-buffer (170 mM NH^+^_4_, 170 mM Cl^−^, 110 μM EDTA, 220 μM Na^+^, 1 mM K^+^, 1 mM HCO^−^_3_) and the ATP concentration was determined using the luciferin-luciferase assay as described below.

### Luciferin-luciferase assay

The ATP Determination Kit (A22066, Invitrogen) was employed for the luciferin-luciferase assay using a modified version of the vendor's protocol. To each well in a 96-well plate, the kit reaction buffer was added to the samples or standard solution provided by the kit at room temperature. The samples and standards was read immediately after in an Enspire 2300 Multilabel Reader (PerkinElmer, Waltham, MA, USA) for the hypotonic stress assay and a Mithras LB940 Multimode Reader (Berthold Technologies, Bad Wildbad, Germany) for measurement of the ATP content of intact vesicles. In the hypotonic stress assay, four different sets of ATP standards were run alongside the samples from each experiment to account for the effects of hypotonic dilution and DMSO, in which bafilomycin A1 was dissolved. In the vesicle assay, two different sets of standards were run to account for the high K^+^ buffer and the lysis-buffer.

### Solutions

The HEPES-buffered salt solution had the following composition, in mM: [Na^+^] 138, [K^+^] 5.3, [Ca^2+^] 1.8, [Mg^2+^] 0.8, [Cl^−^] 126.9, [SO^2−^_4_] 0.8, HEPES 14, glucose 5.6, probenecid 5, pH 7.4 (37°C, 300 mOsmol l^−1^). The Ca^2+^-free solution had the following composition, in mM: [Na^+^] 139, [K^+^] 5.3, [Mg^2+^] 0.8, [Cl^−^] 125.3, [SO^2−^_4_] 0.8, EGTA 1, Hepes 14, glucose 5.6, probenecid 5, pH 7.4 (37°C, 300 mOsmol l^−1^). TE-buffer, in mM: Tris-HCl 10, EDTA 1, pH 7.5. Lysis buffer for vesicle lysis had the following composition, in mM: [NH^+^_4_] 170, [Cl^−^] 170, EDTA 0.1, [Na^+^] 0.2, [K^+^] 1, [HCO^−^_3_] 1. High K^+^-solution had the following composition, in mM: [Na^+^] 14, [K^+^] 125 mM, [Cl^−^] 125, [Mg^2+^] 0.8, [SO^2−^_4_] 0.8, HEPES 14, glucose 5.6, pH 7.2.

Sources of chemicals were: fluo 4-AM and BAPTA-AM (Invitrogen), probenecid, quinacrine from Sigma, and MANT-ATP from Jena Bioscience (Jena, Germany). The solution used in fluo 4 experiments contained 5 mM probenecid to inhibit extrusion of the dye. All experiments were carried out at 37°C, pH 7.4.

The following substances were diluted in HBS: ATP, probenecid, N-ethylmalemide; ethanol: Brefeldin A and ionomycin. The following substrances were diluted in DMSO: bafilomycin A1, cytochalacin B, 18α-glucyrrhetinic acid and nocodazol. The content of vehicle in all experiments did not exceed 0.1% (*v*/*v*), which does not influence the [Ca^2+^] oscillations.

### Statisticcal analysis

All values are shown as the mean ± s.e.m. Statistical significance was determined using the Mann-Whitney-Wilcoxon non-parametric test for comparison of two groups and the One-Way ANOVA followed by a Tukey-Kramer multiple comparison test for comparison of more than two groups. In both cases a *p-value* less than 0.05 was considered significant. The number of observations refers to the number of preparations (independent experiments) analyzed.

## Results

### Spontaneous and stimulated [Ca^2+^]_i_ increase in MDCK cells

**Figure 1:**
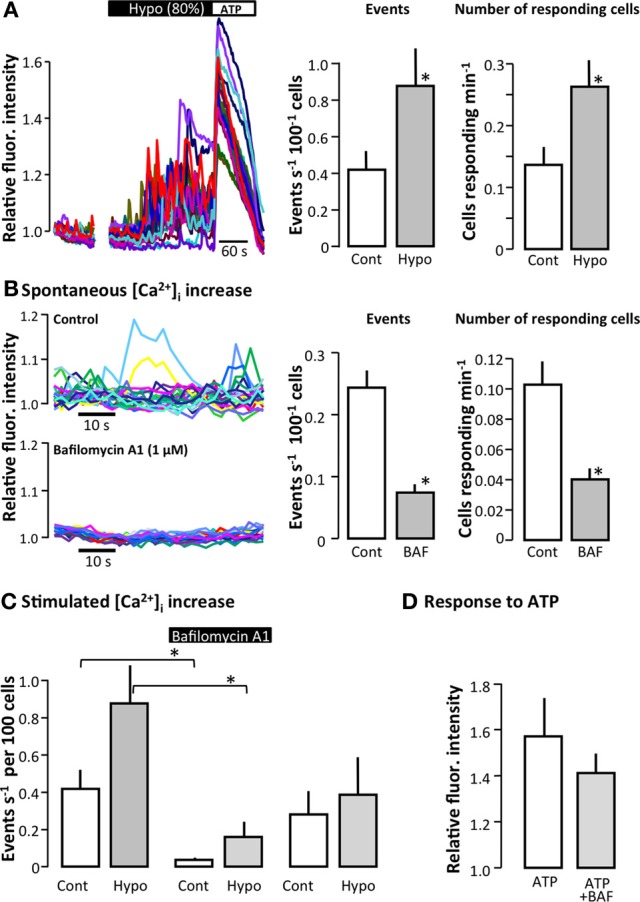
**The effect of bafilomycin A1 on the spontaneous and stimulated [Ca^2+^]_i_ increase in MDCK cells**. **(A)** The left part of the figure shows the original trace of the fluo 4 fluorescence in MDCK cells. The initial part of the trace illustrates the small number of spontaneous [Ca^2+^]_i_ events in confluent MDCK cells during baseline, followed by the effect of reducing osmolality to 80%. The right part of the figure shows the summarized data in terms of the total number of [Ca^2+^]_i_ events greater than 5% above baseline and the number of responding cells; values are mean ± s.e.m. (*n* = 16). **(B)** The left part of the figure shows original traces of spontaneous [Ca^2+^]_i_ events in a control situation and after the addition of bafilomycin A1 (1 μM, 30 min). To the right, the summarized data are shown as the total number of spontaneous [Ca^2+^]_i_ events and the number of responding cells; values are mean ± s.e.m. (*n* = 9). **(C)** The left part of the figure shows the effect of bafilomycin A1 (Baf, 1 μM, 30 min) on the [Ca^2+^]_i_ events induced by reducing the osmolality to 80% of the control. The right part shows the [Ca^2+^]_i_ activity 30 min after return to control solution after washout of bafilomycin A1; values are mean ± s.e.m. (*n* = 15). **(D)** Effect of bafilomycin A1 on the ability of MDCK cells to react to extracellular ATP (100 μM); values are mean ± s.e.m. (*n* = 16). The asterisks indicate statistical significance.

Previously, we have shown that renal epithelial cells spontaneously release ATP. By carefully comparing the spontaneous [Ca^2+^]_i_ oscillations in MDCK cells and perfused renal tubules with extracellular ATP, as measured using the luciferin/luciferase assay, we provided evidence that these spontaneous [Ca^2+^]_i_ oscillations were indicative of the spontaneous release of extracellular ATP by the renal epithelia (Geyti et al., 2008). We used this association to screen for potential ATP release pathways in type 1 MDCK cells, which in our laboratory consist of approximately 90% principal- and 10% intercalated-like cells. We previously established a protocol to detect incremental increases in the [Ca^2+^]_i_ of greater than 5% above baseline (Geyti et al., 2008). Changing to an 80% hypotonic solution on the apical side triggered a significant increase in the number and amplitude of the [Ca^2+^]_i_ oscillations observed in MDCK cells (Figure 1A).

### Bafilomycin A1 reduces spontaneous and stimulated ATP release

**Figure 2:**
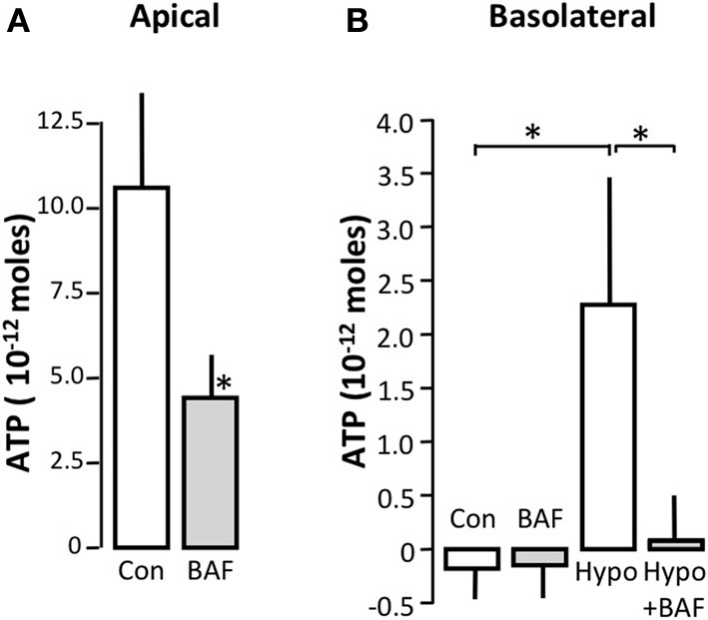
**The effect of bafilomycin A1 on the spontaneous and stimulated ATP release from MDCK cells**. **(A)** Effect of bafilomycin A1 (1 μM) on ATP release from the apical side under baseline conditions. **(B)** Effect of bafilomycin A1 on the ATP release from the basolateral side under baseline conditions and after reducing the osmolality to 50% of the original value on the basolateral side in the absence or presence of bafilomycin A1 (1 μM). The values are shown as mean ± s.e.m. (*n* = 8). The asterisks indicate statistical significance.

Bafilomycin A1 inhibits vesicular H^+^-ATPases, which in addition to maintaining a continuously low intravesicular pH, also provide the driving force for vesicular accumulation of various transmitters such as glutamate, 5-hydroxytryptamine (5-HT), γ-Aminobutyric acid (GABA) (Moriyama and Futai, 1990), and modified amino acids (Al-Damluji and Kopin, 1996). Moreover, the H^+^-ATPases and thus, bafilomycin A1 have previously been demonstrated to be essential for the accumulation of ATP in zymogen granules in the exocrine pancreas (Haanes and Novak, 2010). Figure 1B illustrates the effect of bafilomycin A1 on spontaneous [Ca^2+^] oscillatory activity in MDCK cells as an original trace, the summarized number of events and the number of responding cells. Bafilomycin A1 significantly reduced spontaneous [Ca^2+^]_i_ oscillations in terms of both the total number of events and the number of cells that showed spontaneous [Ca^2+^]_i_ oscillations (Figure 1C). With respect to stimulated [Ca^2+^]_i_ elevations, bafilomycin A1 significantly reduced the [Ca^2+^]_i_ response to hypotonic stress, whereas the response to externally applied ATP was unaffected by the treatment (Figure 1D). We also confirmed that bafilomycin A1 reduced the spontaneous ATP release from the apical side of MDCK cells (*p* < 0.05, Figure 2A). The level of spontaneous ATP-release to the basolateral side of the cells was under the detection-limit in our assay. Therefore the ATP release from MDCK cells was stimulated by hypotonic stress (50%), and this ATP release showed distinct bafilomycin sensitivity (*p* < 0.05, Figure 2B). Bafilomycin A1 did not influence the ATP determination assay, but standard curves were included for all ion-compositions as the ion content has dramatic effect on the assay. These results are consistent with a hypothesis that vesicular acidification is involved in both spontaneous and stimulated [Ca^2+^]_i_ increase.

### Interference with the vesicular release pathway inhibits spontaneous and stimulated [Ca^2+^]_i_ increase

**Figure 3:**
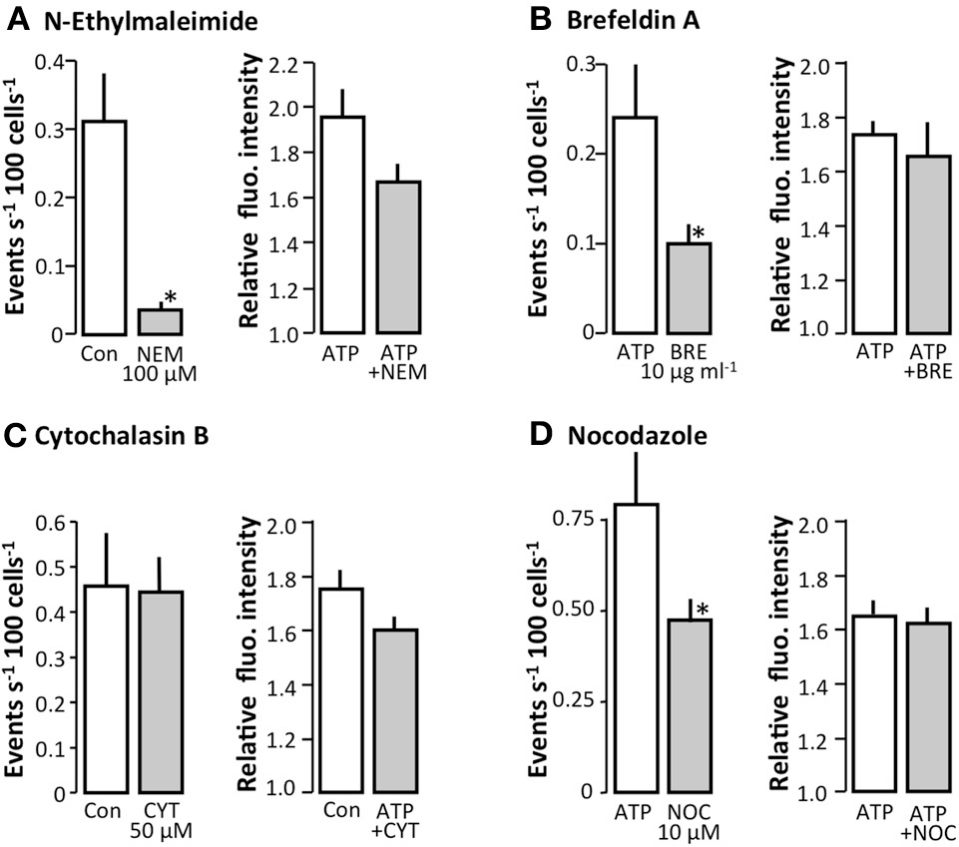
**The effect of various inhibitors of the exocytotic pathway on spontaneous [Ca^2^+]_i_ events in MDCK cells. (A)** The effect of N-ethylmalimide (NEM, 100 μM, *n* = 6), **(B)** Brefeldin A (BRE, 10 μg ml^−1^, *n* = 13), **(C)** Cytochalasin B (CYT, 50 μM, *n* = 5), and **(D)** Nocodazole (NOC, 10 μM, *n* = 7). The left hand side of the figures show the spontaneous [Ca^2+^]_i_ events and the right hand side shows the response to the application of ATP (100 μM). All values are mean ± s.e.m. The asterisks indicate statistical significance.

To investigate if ATP was released by exocytosis, we tested various substances that interfere at different points of the exocytosis pathway. Figure 3 shows the effect of *N*-ethylmaleimide (NEM), brefeldin A, cytochalasin B, and nocodazole on spontaneous [Ca^2+^]_i_ increments, shown as the number of events. NEM interferes with the ability of vesicles to dock to the plasma membrane by inhibiting the function of Soluble *N*-ethylmaleimide-sensitive factor Attachment Protein receptors (SNAREs) and Soluble *N-ethylmaleimide-sensitive* factor Attachment Proteins (SNAPs), which are required for this process. We observed that NEM (100 μM) and significantly reduced the spontaneous [Ca^2+^]_i_ oscillations in MDCK cells (*p* = 0.0022). Similar results were found with brefeldin A (10 μg ml^−1^), which interferes with the vesicular movement of proteins from the ER to the Golgi apparatus and within the Golgi apparatus (*p* = 0.042). Nocodazole (10 μM) inhibits vesicles mobility by interfering with microtubule polymerization, along which the vesicles are transported (Bjaelde et al., 2011). When nocodazole (10 μM) was added, we observed a significant inhibition of spontaneous [Ca^2+^]_i_ oscillations in MDCK cells (*p* = 0.017). However, the number of responding cells per min was not affected by nocodazole. Cytochalasin B (50 μM), which inhibits the assembly of actin filaments, had no effect on spontaneous [Ca^2+^]_i_ oscillations. None of the drugs interfered with the ability of the cells to react to agonist stimulation, as indicated by the response of the cells to the positive control ATP (100 μM, see the right hand side of Figures 3A–D).

**Figure 4:**
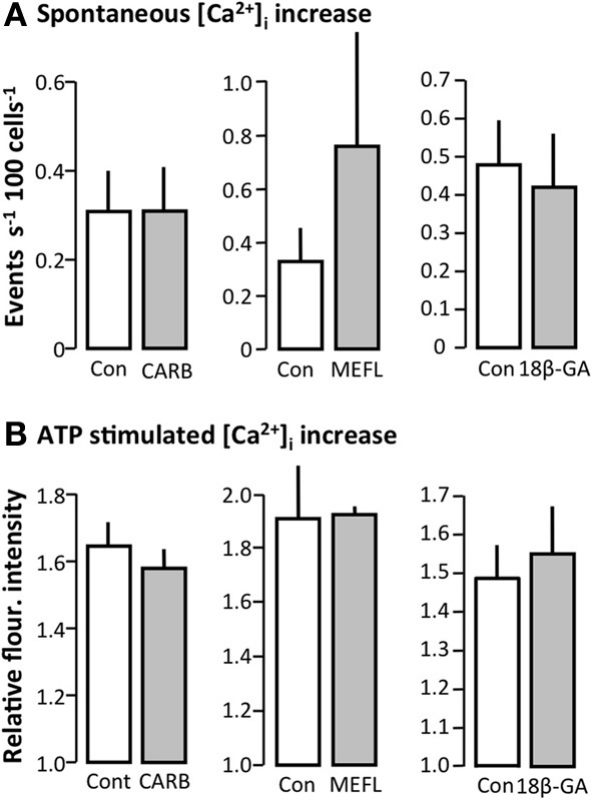
**Effect of gap junction and pannexin inhibitors on spontaneous [Ca^2+^]_i_ events in MDCK cells**. **(A)** Carbenoxelone (CARB, 100 μM, *n* = 7), mefloquine (MEFL, 100 μM, *n* = 2) and 18β-Glychyrritinic acid (18β-GA, 50 μM, *n* = 12). **(B)** Corresponding reactivity to ATP (100 μM). All values are given as mean ± s.e.m.

We also screened for effect of blocking connexin hemi-channels and pannexins to determine the roles of these pathways in the release of ATP. Carbenoxolone (100 μM) and 18β-glycyrrhetinic acid (50 μM) had no effect on the spontaneous [Ca^2+^]_i_ events, whereas mefloquine (100 μM) promoted an increase in the spontaneous [Ca^2+^]_i_ events (Figure 4). In this context, it should be emphasized that [Ca^2+^]_i_ imaging requires addition of probenecid to inhibit export of probe via multidrug resistance-associated protein 2. Probenecid is also known to inhibit pannexins, but has no effect on connexins-hemichannels at 5 mM (Silverman et al., 2008). Probenecid (5 mM) did not inhibit either the spontaneous or the stimulated (hypotonic) ATP release from MDCK cells as measured by the luciferin-luciferase assay (data not shown); rather, probenecid induced a slight increase in the spontaneous ATP release from the cells.

### siRNA-mediated silencing of the canine vesicular docking protein SNAP-23 interferes with vesicular release

**Figure 5:**
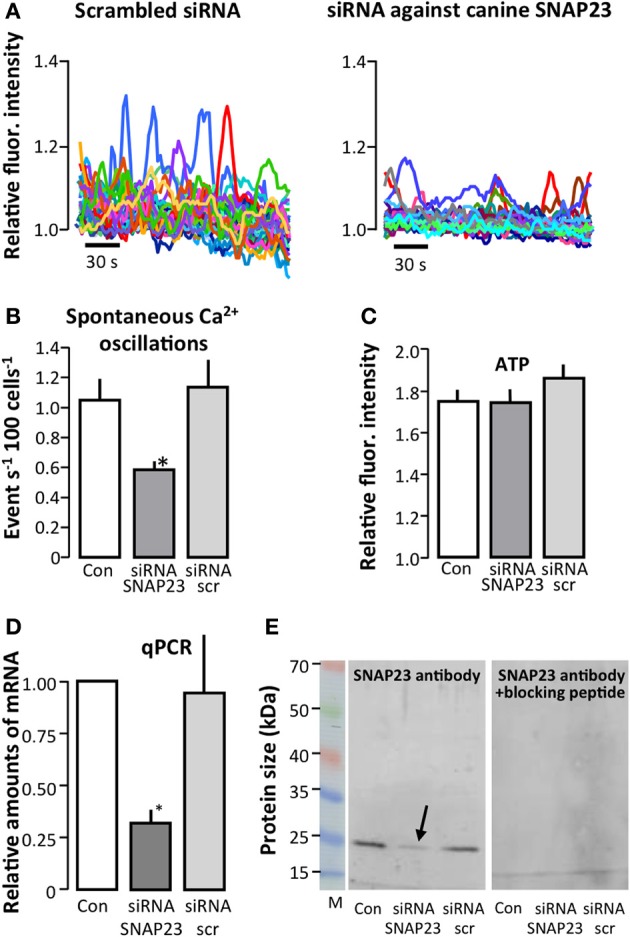
**Effect of siRNA-mediated knockdown of canine SNAP-23 in MDCK cells**. The effect of transfection of MDCK cells with scrambled siRNA (siRNA scr) or siRNA directed against SNAP-23 on spontaneous [Ca^2+^]_i_ events; representative original traces shown in **(A)** and **(B)** shows the summarized data as number of events s^−1^ as mean ± s.e.m. (Con: *n* = 12, siRNA SNAP-23: *n* = 9, siRNA scr: *n* = 8). **(C)** The effect of knocking down canine SNAP-23 on the ability of MDCK cells to respond to ATP (100 μM, Con: *n* = 12, siRNA SNAP-23: *n* = 9, siRNA scr: *n* = 8). Please note that preparations, which did not respond to extracellular ATP were not included in the analysis. **(D,E)** Confirmation of siRNA-mediated knockdown of SNAP-23 by qPCR **(D)** and immunoblotting **(E)**. The arrow indicates the reduced protein expression of SNAP-23 (~25 kDa, expected size ~23 kDa) in the lysate of cells transfected with siRNA directed against SNAP-23 (left). The control blot incubated with primary antibody, which had been pre-incubated with a SNAP-23 peptide is shown to the right. Con: *n* = 4, siRNA SNAP-23: *n* = 4, siRNA scr: *n* = 3. All experiments were performed at 48 h after transfection of the MDCK cells. The asterisks indicate statistical significance.

So far, our data support the hypothesis that ATP may be released by vesicular fusion from renal epithelial cells. To further test this notion, we silenced canine SNAP-23, a protein which is an important part of the docking mechanism responsible for docking of vesicles to the target membrane (Ravichandran et al., 1996; Wong et al., 1997). Figure 5A shows representative original traces of the changes in [Ca^2+^]_i_ using scrambled siRNA (left) or siRNA directed against SNAP-23 (right). Transient knockdown of the SNAP-23 protein in MDCK cells reduced the spontaneous [Ca^2+^]_i_ oscillations significantly to 47% at 48 h after siRNA transfection, whereas transfection with the siRNA had no effect compared to the control mock-transfected cells (Figure 5B). The transfection process did not affect ability of the cells to react to 100 μM ATP (Figure 5C). Quantitative rtPCR confirmed a significant reduction in relative amounts of mRNA of 56% compared to control was seen (Figure 5D), and immunoblotting demonstrated that SNAP-23 protein expression was reduced by ~70% (arrow in Figure 5E) in SNAP-23 siRNA transfected cells compared to scrambled control siRNA transfected cells.

### Observation of direct vesicle-fusion using TIRF microscopy

**Figure 6:**
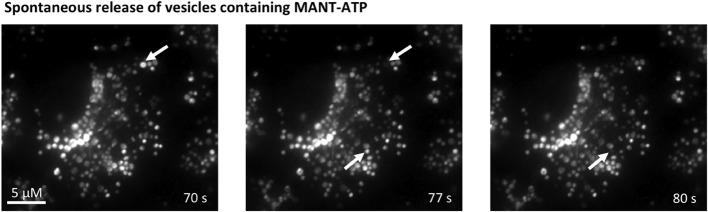
**Spontaneous vesicular fusion in MDCK cells**. Total internal reflection fluorescence (TIRF) microscopy of MDCK cells loaded with MANT-ATP (25 μM, 5 h, 37°C). Arrows indicate vesicles that abruptly disappeared from the evanescence field.

**Figure 7:**
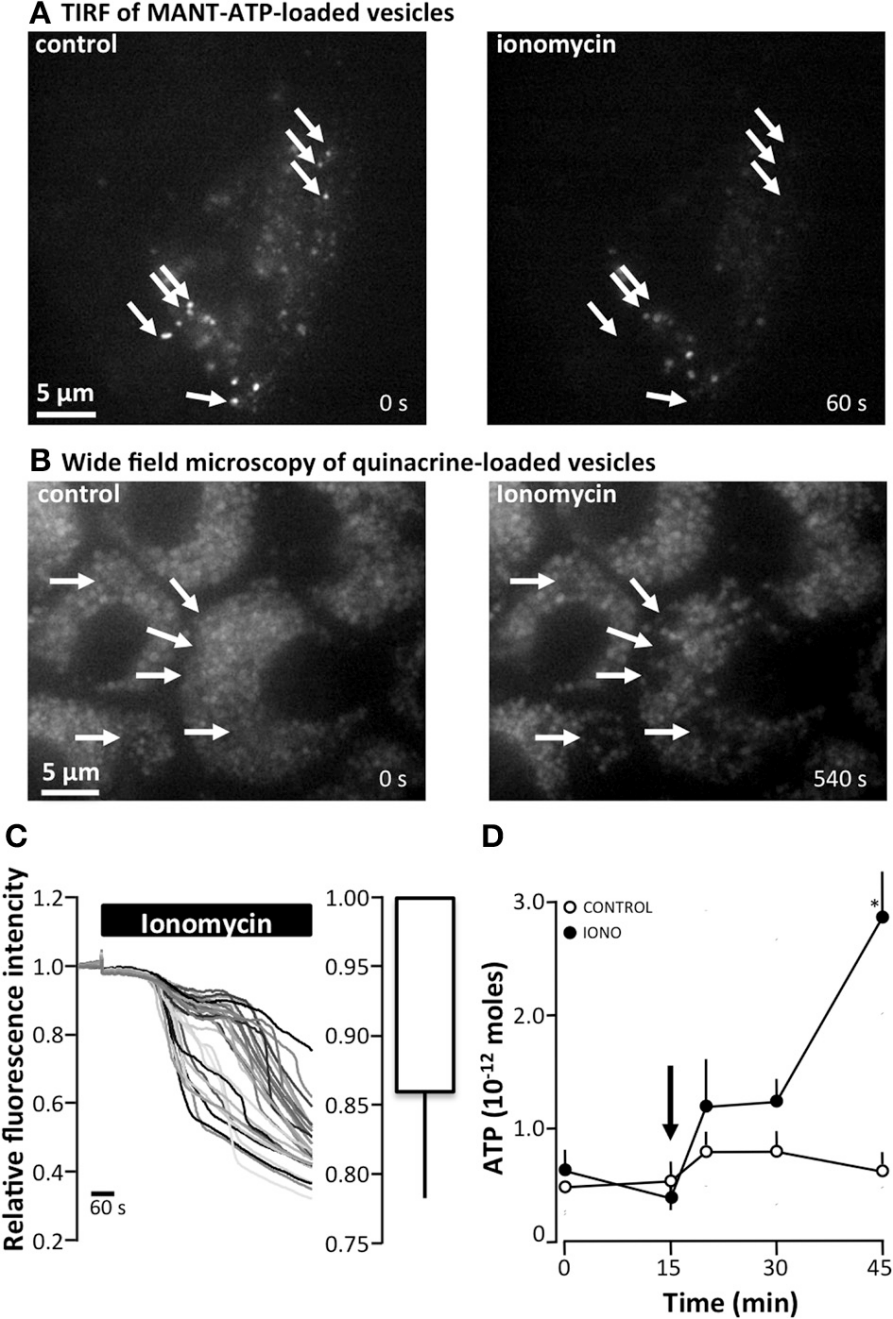
**The effect of ionomycin on ATP release and vesicular fusion in MDCK cells (A) total internal reflection fluorescence (TIRF) microscopy of MDCK cells loaded with MANT-ATP (25 μM, 5 h, 37°C) showing vesicular fusion after the addition of ionomycin (1 μM)**. Arrows indicate vesicles that abruptly disappeared from the evanescence field. **(B)** Ionomycin (1 μM)-induced vesicular fusion as indicated by wide-field fluorescence microscopy of MDCK cells loaded with quinacrine (5 μM, 30 min, 37°C). Arrows indicate vesicles that display an abruptly drop in fluorescence. **(C)** Right: Original trace of the effect of ionomycin (1 μM, 37°C) on the relative fluorescence intensity of quinacrine-loaded MDCK cells. The left panel present the mean ± s.e.m. from 6 experiments. **(D)** ATP release from MDCK cells induced by apical application of ionomycin (IONO, 1 μM). The arrow indicates the addition of ionomycin or the control solution; the mean ± s.e.m. values are presented (*n* = 10). The asterisk indicates statistical significance.

To be able to observe direct vesicle-fusion with the plasma membrane we used two different dyes: quinacrine, which is known to accumulate in acidic vesicles and MANT-ATP for TIRF microscopy. The images obtained the staining patterns of the two dyes seemed very similar, even though quinacrine appeared to stain more vesicles than MANT-ATP. It was not possible to verify whether the two probes stained the same population of vesicles, because of overlapping excitation and emission spectra of the dyes. Moreover, MANT-ATP bleaches very rapidly, making this probe unsuitable for longer time-lapse studies. Using both dyes, it was possible to observe spontaneous disappearance of vesicles (arrows in Figure 6) or more consistently after stimulation with the Ca^2+^ ionophore ionomycin (arrows in Figures 7A,B). In Figure 7A, the arrows indicate vesicles that disappeared abruptly between one frame and the next (sampling rate 1 Hz). With identical intensity histogram profiles for the earlier and later pictures, it is hard to distinguish the vesicle that disappeared rapidly from the evanescence field from the ones that have bleached. By narrowing the histogram, one can still see the remaining bleached MANT-ATP vesicles and confirm that they have not disappeared from the evanescence field. On the other hand, quinacrine was bleached at a negligible rate. Using wide-field fluorescence, we estimated what proportion of the overall quinacrine fluorescence was reduced in response to ionomycin. Figure 7C shows the initial stable baseline level of quinacrine fluorescence. After addition of ionomycin, the overall fluorescence is reduced significantly over time. Each line in the original trace on the left-hand side of Figure 7 represents a region of interest (ROI) placed over the quinacrine-loaded vesicles in the cytosol of a single cell. The right-hand side of the figure summarizes the average reduction in the total fluorescence intensity 8 min after the addition of ionomycin. Figure 7D illustrates that ionomycin (1 μM) did stimulate detectable release of ATP to the extracellular space measured by the luciferin/luciferase assay.

**Figure 8:**
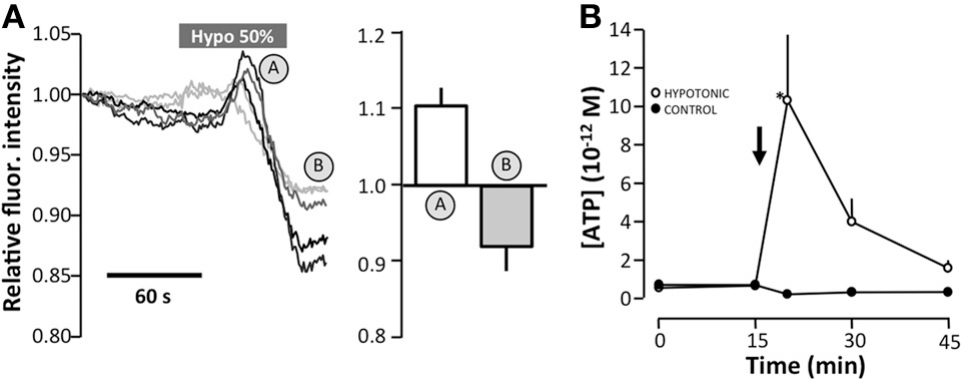
**The effect of hyposmotic stress on vesicular fusion of quinacrine-loaded vesicles by TIRF microscopy of MDCK cells**. **(A)** Original traces of the total fluorescence intensity of 5 cells as determined by TIRF microscopy. The mean ± s.e.m. values are shown on the right as *n* = 17. **(B)** Effect of apically-induced hypotonic stress (50%) on the level of extracellular ATP. Data are presented as the mean ± s.e.m. (*n* = 10). The asterisk indicates statistical significance.

Moreover, we observed that reducing the osmolality also accelerated the disappearance of quinacrine-loaded vesicles. Figure 8A shows the average change in fluorescence intensity measured by TIRF microscopy. As before, each original trace represents the average fluorescence intensity of the cytoplasm of a single cell. Reducing the osmolality initially increased the overall fluorescence intensity, as apparently more vesicles were recruited into the evanescence field, where after the overall fluorescence intensity subsequently reduced. The impact of reducing the osmolality on the overall fluorescence intensity was of a shorter duration and less extensive than the reduction observed in response to ionomycin (1 μM). Figure 8B shows the corresponding release of ATP observed after reducing the osmolality apically as measured by the luciferin/luciferase assay.

### Isolation of intact vesicles and determination of their ATP concentration

To investigate whether or not the quinacrine-loaded vesicles actually contained ATP, we isolated intact vesicles using the EMBL cell cracker and FACS sorting. The cell cracker mechanically disrupts the cell membrane, and by using a buffer resembling the cytosolic environment, the vesicles should remain intact. The isolation of intact vesicles in the cell lysates was confirmed by microscopy (data not shown), before the vesicles were sorted from the rest of the cell debris by FACS. The ATP concentration of the vesicles was calculated to be around 8.5 mM. This value was calculated based on the average diameter of the vesicles (0.9 μm; measured from time-lapse microscopy), together with the FACS data on the number of sorted vesicles per ml and the concentration of ATP, determined by the luciferin-luciferase assay. This experiment was performed twice and 8.5 mM is the average value obtained.

## Discussion

ATP-mediated signaling has a major impact on renal function. Generally, epithelial ATP release will reduce the transepithelial transport (Kishore et al., 1995; Bailey, 2004; Pochynyuk et al., 2008; Marques et al., 2012) directly via activation of P2 receptors on the epithelium itself or indirectly by reducing the demand for transport through the tubulo-glomerular feedback mechanism via A1_a_ receptors (Sun et al., 2001). Consistent with this, mice deficient in one of the most important P2 receptors in renal epithelial cells, the P2Y_2_ receptor, exhibit hyper-reabsorption of Na^+^ in the collecting duct and hypertension. ATP is known to be released from renal epithelial cells in response to mechanical perturbations, such as an increased fluid flow rate (Praetorius and Leipziger, 2009) and hypotonic swelling (Boudreault and Grygorczyk, 2004), and is also released in response to AVP (Odgaard et al., 2009).

As a result of the impact that P2 receptor activation has on kidney function, it is unsettling that so little is known about the mechanism of ATP release. Cx30 has been suggested to function as an ATP channel in distal tubular intercalated cells (Sipos et al., 2009), where cx30 is specifically expressed in the apical domain of the cells (McCulloch et al., 2005). Although cx30 is clearly able to form gap junctional channels (Valiunas et al., 1999; Valiunas and Weingart, 2000), the subcellular localization of this protein in the intercalated cells suggests that gab junction formation is not cx30 main function in this cell type. In the collecting duct, the spontaneous and stimulated ATP release are intimately associated with the expression of cx30 (Sipos et al., 2009), which suggests that a hemichannel configuration of cx30 may serve as the main pathway of ATP release. These data are supported by a previous study of cultured HEK cells overexpressing cx30 (Liang et al., 2011), which took advantage of the feature that the open state probability of connexin hemi-channels increases as [Ca^2+^]_e_ is reduced. Chelating extracellular Ca^2+^ triggered the cx30-expressing HEK cells to release more ATP compared to wild type, whereas the ATP release at normal Ca^2+^ concentrations was similar in cx30 expressing and wild type HEK cells (Liang et al., 2011). This finding supports the notion that cx30 is an important pathway for ATP release in renal epithelial cells.

As only a select sub-fraction of renal epithelial cells express cx30, cx30 cannot be the only pathway for ATP release. Therefore, we investigated the possibility of alternative pathways of ATP release in renal epithelia. We addressed this issue using a screening procedure for constitutive ATP release in MDCK cells. We found that substances that interfere with vesicular loading and release significantly reduced the spontaneous increases in [Ca^2+^]_i_, which we monitored as a proxy for ATP release. This [Ca^2+^]_i_ assay has the clear disadvantage that it does not directly measure ATP release; however, on the other hand, the method is very reliable and avoids the interference resulting from changes in the cellular environment or the effect of pharmacological substances on the enzymatic reactions in the luciferein/luciferase method. We have previously validated this method and demonstrated that the degree of spontaneous [Ca^2+^]_i_ increase is direct result of constitutive ATP release in both MDCK cells and freshly isolated renal epithelial cells (Geyti et al., 2008). Via this method, we found that bafilomycin A1 significantly reduced both spontaneous and hypo-osmotically-induced [Ca^2+^]_i_ increase in MDCK cells. Moreover, we found that substances that potentially interfere with vesicular release (brefeldin A, nocodazole and NEM) reduced the spontaneous [Ca^2+^]_i_ events. However, pannexin and connexin blockers, did not have any detectable effect on the spontaneous [Ca^2+^]_i_ events. The effect of pharmacological interference with vesicular release was substantiated by siRNA-mediated knockdown of the tSNARE SNAP-23 in MDCK cells. Transient knockdown of SNAP-23 reduced SNAP-23 mRNA and protein expression by approximately 56 and 70%, respectively, at 48 h. In parallel experiments, spontaneous [Ca^2+^]_i_ oscillations were significantly reduced to 47% in cells transfected with the SNAP-23 siRNA compared to cells transfected with a scrambled siRNA. Using bafilomycin A1 as a representative pharmacological inhibitor of vesicular release pathway, we confirmed that inhibition of the vesicular release pathway also reduced constitutive and osmotically-induced ATP release as measured with the luciferin-luciferase assay.

To investigate whether it was possible to detect vesicular release in MDCK cells, we used various approaches to stain ATP-containing vesicles. Quinacrine has been used as a marker for ATP containing vesicles on several occasions (Bodin and Burnstock, 2001; Sorensen and Novak, 2001; Akopova et al., 2012). Quinacrine has also been shown to accumulate in acidic vesicles and has been used as a marker of lysosomes (Bastos et al., 1966). Staining the cells with acridine orange, which has not been associated with ATP content, produced a staining pattern similar to that of cells stained with quinacrine (Bjaelde et al., 2011). Moreover, bafilomycin A1, an inhibitor of vesicular H^+^-ATPases, has also been shown to prevent the accumulation of quinacrine in intracellular vesicles (Marceau et al., 2009), either as a result of a reduced vesicular pH directly or, in light of our results, as a bafilomycin A1-induced lack of ATP in the granules. Regardless of its shortcomings, quinacrine is at present a reasonable tool to address whether MDCK cells are able to release vesicles, which may potentially contain ATP. To substantiate the release of ATP-containing vesicles, we also used the fluorescently-labeled ATP molecule MANT-ATP, with the expectation that the cell would process this probe in the same manner as endogenous ATP. After prolonged incubation, we observed that MANT-ATP accumulated in vesicles in a similar manner to quinacrine.

We have attempted to confirm whether the quinacrine-stained vesicles actually contained ATP. After mechanically disintegrating the MDCK cells, we used FACS to sort the cellular component on the basis of fluorescence and size, and selectively recovered the smaller particles with a high fluorescence signal when excited at 488 nm. From the number of vesicles retrieved and the ATP content of the vesicle lysate, the vesicles were determined to contain approximately 8.5 mM ATP, compared to the concentration of ATP in the cytosol of 1–3 mM (Ainscow and Rutter, 2002), the concentration of ATP in the vesicles was only slightly higher. We have good reason to assume that the value of 8.5 mM is an underestimation. The vesicles were measured to have an average diameter of 0.9 μm, which is likely to be an overestimation as a result of the fluorescent blur and the limitations of the resolution in the x-y plane. More importantly, if H^+^-ATPases are required for the continuous accumulation of ATP in the vesicles then this activity was not supported after the cells were disintegrated and the ATP surrounding the vesicles was washed away, despite the fact that we tried to reduce the leakage of ATP from the vesicles by keeping the preparations on ice until FACS.

Using either quinacrine or MANT-ATP, we were able to observe spontaneous drops in vesicular fluorescence using TIRF microscopy. The sudden fall in vesicular fluorescence were taken as account for direct vesicular fusion with the plasma membrane. This technique has been employed by others to visualize the release of ATP-containing vesicles from lung epithelial cells (Akopova et al., 2012). To stimulate exocytosis, we used either ionomycin or hypotonic stress. Ionomycin is known to induce sustained elevations in [Ca^2+^]_i_, and does provoke a substantial release of ATP by MDCK cells. Ionomycin also promoted increased vesicle fusion with the plasma membrane; at longer incubations this effect was so substantial that it was visible as a decrease in the overall fluorescence of the cells in wide-field microscopy. The pattern was slightly different when vesicular release was induced by hypotonic stress. When the osmolality of the solution was reduced, we initially observed increased recruitment of vesicles to the membrane, which was observed as an increase in fluorescence using TIRF microscopy. Following this initial recruitment, subsequent degranulation was observed as sudden drops in vesicular fluorescence. These observations are consistent with the process of vesicular release as one possible mechanism to induce ATP release from renal epithelial cells.

In terms of ATP release through pannexins and connexins, we were unable to detect any evidence for a significant contribution of these pathways to the ATP release from MDCK cells. In many ways, these results are not surprising as the cx30-mediated ATP release is mainly observed in intercalated cells, and in our laboratory, the MDCK type 1 culture contained fewer than 10% intercalated cells, determined as the proportion of non-ciliated cells in the confluent state. However, MDCK type 1 cells, do express connexins, which can form functional gap-junctions between the cells (Cereijido et al., 1984). Regardless of the numerous reports on connexin-hemi-channels as ATP release pathway, the physiological relevance of this pathway has been questioned with regards to the properties of the channel for stimulated ATP release. The main criticism is the inverse dependency of connexin-hemichannels/gap junctions on [Ca^2+^]_i_, whereas potential ATP release pathways would require increases in [Ca^2+^]_i_ to activate the given pathway (for review see Saez et al., 2003). Connexons are generally known to be closed at physiological [Ca^2+^]_e_, a feature, which is in many respects not an ideal one for stimulated ATP release (Li et al., 1996; Contreras et al., 2003; Locovei et al., 2006). This controversy has led to the suggestion of pannexins, which do not form gap-junctions, as a pathway for ATP release (Locovei et al., 2006). Pannexin 1-mediated ATP release was initially demonstrated in erythrocytes (Locovei et al., 2006) and was subsequently shown to be important in many other tissues, including respiratory (Seminario-Vidal et al., 2011) and renal epithelia (Hanner et al., 2012). Taken together, we find it unlikely that connexins and pannexins contribute significantly to the ATP release in this cell culture model of renal epithelia. Whether this finding can be extrapolated to renal epithelial cells in general remains to be established.

In summary, we report that both the spontaneous and stimulated ATP release can be mediated by vesicular release in MDCK cells. Our results are consistent with vesicular release as the principle pathway for ATP release in this renal epithelial cell model.However, the predominant mechanisms of nucleotide release in different segments of the intact renal tubules require further investigation. Our present data suggest that vesicular release of ATP should be considered as a viable possibility in the kidney.

### Conflict of interest statement

The authors declare that the research was conducted in the absence of any commercial or financial relationships that could be construed as a potential conflict of interest.
